# Barriers and facilitators of school-based obesity prevention interventions: a qualitative study from the perspectives of primary school headteachers

**DOI:** 10.1186/s41043-024-00713-1

**Published:** 2024-12-18

**Authors:** Mohamed Saleh, Maryam Ba-Break, Asma Abahussin

**Affiliations:** 1 Leeds Institute of Health Sciences, International Health, University of Leeds, Leeds, UK; 2 Leeds Institute of Health Sciences, University of Leeds, Leeds, UK; 3 Department of Biomedical Technology, College of Applied Medical Sciences, King Saud University, P.O Box 10219 Riyadh, Saudi Arabia

**Keywords:** Childhood obesity, Childhood obesity prevention, School meals, Extracurricular activities, And parental awareness

## Abstract

**Background:**

Childhood obesity is a growing global health issue. The World Health Organization identifies obesity as a significant risk factor for Non-Communicable Diseases and considers it a pandemic. This study aimed to investigate headteachers' perspectives and practices concerning childhood obesity prevention interventions in primary schools highlighting the barriers and facilitators for those interventions.

**Methods:**

This qualitative study used a phenomenological approach and semi-structured interviews with headteachers of primary schools in the West Yorkshire area, UK. The collected data was transcribed and analysed using inductive thematic analysis.

**Results:**

A total of 32 interviews with headteachers were conducted. The data indicated that interventions meant to prevent childhood obesity in schools are influenced by a range of barriers and facilitators that are organized under the following seven key themes: (1) staff perception of obesity prevention at school; (2) school policies on eating at schools; (3) School curriculum on healthy diets and physical activities; (4) role models at school; (5) partnership with parents; (6) extra-curricular activities on healthy diets and physical activities; (7) School capacity and resources.

**Conclusions:**

Childhood obesity prevention interventions vary across UK schools depending on staff and head teachers' beliefs, awareness, commitments, school resources, parents' involvement and parents’ awareness, income, and culture. The study suggests raising awareness of childhood obesity among parents and staff, involving external partners like school nurses for training, making all primary pupils eligible for free meals, and providing schools with guidance on securing government funding. It provides a foundation for improving school-based strategies that indirectly contribute to better health outcomes for children.

## Introduction

Childhood obesity is a growing global health issue, with 124 million children and adolescents being obese 6% of girls and 8% of boys worldwide [35]. In England, childhood obesity rates have steadily increased, with a third of children leaving primary school overweight or obese [10]. West Yorkshire has some of the highest obesity rates among school children [26].

The World Health Organization (WHO) identifies obesity as a major risk factor for Non-Communicable Diseases (NCDs) and considers it a pandemic [23]. Childhood obesity is linked to adult obesity, which can lead to severe health complications like hypertension, inflammation, and insulin resistance, with type 2 diabetes increasingly affecting adolescents [22, 38]. The normalization of obesity is driven by complex societal, behavioural, and environmental factors [5], prompting calls for a systems-based approach to prevention [8]. Unhealthy diets and sedentary lifestyles contribute to rising obesity rates [1], and schools play a crucial role in prevention efforts, especially in shaping long-term habits [19] The effectiveness of school-based interventions in preventing childhood obesity has been identified in some studies in various contexts as they provide young children with the necessary knowledge, attitude, and skills to initiate healthy eating and physical activity at an early age, maintain them during school years, and increase the likelihood of maintaining these healthy lifestyles after school age [6, 29]. However, parents and community involvement and consulting school staff are key to ensuring appropriateness, acceptability, and sustainability [20], especially among the poor and disadvantaged population [27].

Among England, West Yorkshire has high obesity rates among school children [26] and there is limited research exploring how schools' role in obesity control could be enhanced in West Yorkshire. Therefore, t**his study aims** to explore the current role of primary schools in preventing childhood obesity, highlighting the barriers and facilitators from the perspective of head teachers in West Yorkshire, UK. Headteachers play a key role in decision-making and resource allocation, as well as in fostering a culture that supports healthy behaviours among students [9]. Thus, their input is critical for understanding and addressing the challenges specific to the school setting. This study could be followed by another study exploring the views of parents and children toward better intervention implementation.

## Methods

### Study design

This qualitative study used a phenomenological approach to collect data through semi-structured in-depth interviews with Headteachers of primary schools in the West Yorkshire area, UK.

### Sampling and participants recruitment

This study used a purposeful sample of primary school headteachers, typical for phenomenological research focusing on in-depth analysis rather than broad generalization [3]. The sample included diverse criteria such as gender, experience, and school location, including areas with higher obesity rates [25, 26, 36]. Participants were recruited through local authority websites, and emails with study details and consent forms were sent. All schools within the study area were informed of their eligibility for recruitment, but only those who consented were included. Table 1 describes the characteristics of the head teachers who agreed to participate in the study and were interviewed. Females and schools in urban areas represented nearly half of the sample. University of Leeds Ethics-Committee approval was obtained for this study.

**Table 1:** Sample characteristics

Characteristics	Number (total = 32)
Gender	
Female	15
Male	17
Experience as headteacher (years)	
1–5	7
6–10	13
11–15	9
16–20	3
School location	
Rural	8
Urban	17
Deprived Area (yes)	7

### Data collection method

This study used a semi-structured interview to explore headteachers' perceptions. Focus Group Discussions (FGDs) were excluded due to their focus on group dynamics, as the sensitive topic required private, confidential discussions [14, 31]. A semi-structured interview guide with open-ended questions encourages participants to elaborate freely on their experiences, allowing an inductive exploration of "how" and "why" events occurred [24]. A flexible guide was created for this study and piloted for effectiveness.

Interviews were conducted between May 2023 and April 2024 at convenient times and locations for participants, lasting 45–60 min. Most were audio-recorded and transcribed with consent, and handwritten notes were taken when the recording was refused. Key findings and nonverbal cues were noted to ensure reliability [14]. Each interview was analysed before the next, with reflective questions added to guide further investigation.

### Data analysis

The data from this exploratory study was analysed using inductive thematic analysis, suitable for phenomenological research [2, 21]. Interviews were conducted by MS until no new themes or insights emerged from the participants. This was monitored through ongoing thematic analysis during data collection. Once additional interviews stopped to contribute new information to the identified themes, we concluded that saturation had been reached [7]. Data saturation was reached after 32 interviews. MS led the inductive analysis, with all authors systematically identifying patterns and initial themes. Regular discussions among the research team facilitated the agreement on themes and sub-themes, with consensus reached through iterative refinement and validation of the data. We followed the steps used by Creswell and Poth [11] and Guest et al. [17] for conducting the thematic analysis. The steps included (1) transcribing the data, (2) exploring and grouping potential codes, (3) categorising data into key themes and subthemes, (4) identifying relationships between themes, and (5) explaining the observed patterns and their implications. NVivo software V.10 was used for the analysis.

To mitigate investigator bias, the research team employed reflexive journaling throughout the study to ensure ongoing awareness of personal assumptions and perspectives. Additionally, we engaged in regular peer debriefing sessions to discuss findings and interpretations, enhancing the trustworthiness of the data analysis. To address selection bias, participants were recruited using purposive sampling based on predefined criteria to ensure a diverse representation of relevant perspectives, while steps were taken to minimize overrepresentation of any particular subgroup.

A systematic process was used to identify and classify facilitators and barriers. Initially, all investigators reviewed the data independently to identify potential facilitators and barriers. The findings were then discussed in a series of team meetings to resolve discrepancies and achieve consensus. With this collaborative approach, we ensured that the classification reflected a comprehensive understanding of the data, incorporating perspectives from all members of the research team.

## Results

In total, 32 headteachers were interviewed, of whom around 47% were female, and their years of experience as headteachers ranged between two and twenty years. Almost half (17/32) of the included schools were based in urban areas while 22% of the included schools were located in deprived areas as per the UK Index of multiple deprivation which defined these as those that lack the basic necessities of life, society, and proper growth and development in seven domains including Income, Employment, Education, skills and training, Health and disability, Crime, and Barriers to housing and services [12]. See Table 1 for the sample characteristics details.

Inductive analysis of the data collected from primary school headteachers on their perspectives on school-based obesity prevention interventions revealed the following seven themes:

1. Perception of obesity prevention at school
2. School policies on eating at schools
3. School curriculum on healthy diets and physical activities
4. Role models at school
5. Partnership with Parents
6. Extra-curricular activities on healthy diets and physical activities
7. School capacity and resources

1. Perception of obesity prevention at school

It was found that primary school headteachers underestimate obesity prevalence and misunderstand who should be targeted for prevention. Most participants believe obesity interventions are limited to obese and overweight children. Many headteachers assume obesity prevention is not a priority in their schools because childhood obesity is not prevalent.

“The number of obese schoolchildren is not as high as you think.” P2

Headteachers at mixed-ethnic schools said some cultures encourage unhealthy lifestyles. They stressed the importance of addressing obesity among specific ethnic groups. However, targeting overweight children and parents of specific ethnicities is sensitive.

“Families' lifestyles and eating habits affect children of certain ethnicities. It is sensitive to discuss this with their parents, but what else can be done?” P30

2. School policy on eating at schools

We found that schools must follow government policies that emphasise balanced and healthy meals. School menu options include at least three options to accommodate pupils' preferences and dietary restrictions. Local councils review the menu and provide advice to ensure it is balanced and healthy. Students often dislike vegetables and fruits in school meals.

“Children are told to take vegetables and fruits at school... We encourage them, but can't force them... Sadly, many don't eat them, or just eat a little, then toss the rest... Parents or free school meals fund it, but it's a waste!” P25

Schools in West Yorkshire use a similar plate size to monitor portion sizes in school meals. However, children may choose to eat more food if it is available.

“There should be the same size of plates in all schools in the area since school meals are fixed across the area... However, if they have more food to eat at the end of lunch break, they can take more...we know who wants more and who doesn't finish.” P12

For children in key stage one (the first three grades), school meals are free; however, after that, parents are required to pay as free school meals are restricted to pupils of low income. Parents who cannot afford school meals pack their children's lunches since they are not eligible for free meals. Managing packed lunches is challenging because parents provide what they believe is appropriate, affordable, and convenient.

“School meals are expensive for many parents especially those with more than one sibling” P6

It is common for some children from diverse backgrounds to dislike school meals because they are used to certain foods at home, so they pack lunches instead.

“In my experience of 16 years, many parents expect school meals to meet their expectations and make their kids full. It's okay to be unhealthy and imbalanced. Many parents refuse the school's input on how kids should be fed, so they pack lunches.”P9

Monitoring the portion size in packed lunches is impossible and sensitive to be dealt with at school.

It's embarrassing to tell kids they have too much food... Schools could limit portion sizes and introduce healthier options... Parents might object, saying it's not school business... Advocating parents might take courage and time....”P15

School policy differs from school to school regarding the permitted contents of packed lunches. Sweets, chocolate, fries, crisps, and fizzy drinks are frequently banned at school. Many schools have trouble enforcing strict policies and persuading parents to pack healthy lunches for their children. Some schools offer non-financial rewards to students who bring healthy lunches, aiming to encourage healthy eating habits and influence parents' choices.

“Initially, we send letters to parents and explain in assemblies what food is allowed in lunchboxes... Parents are notified if their child has chocolate, crisps, or sugary sweets... Parents are reminded not to get offended when letters are sent to specific kids...” P14

“Despite our best efforts, some parents insist their children eat whatever they want regardless....”P3

“Sometimes, certificates, stickers, and rewards for those who bring fruits and vegetables rather than chocolate or crisps in their lunch boxes... motivate children to eat healthier”.P31

On the other hand, some schools do not have a restrictive policy and believe it is not the school's responsibility to control children's lunches. *“For peace of mind, parents pack whatever food their children enjoy in their lunchboxes, healthy or not… We don't pay for their food, so we can't tell them what to pack.” P10.*

“Lunchbox meals should be decided by parents without pressure from schools... children bring and eat what their parents can afford.”P22

3. School curriculum on healthy diets and physical activities

Primary schools in the UK require children to participate in physical activity (PA) once a week as part of their physical education (PE) curriculum. PE classes are mandatory and taught by trained teachers.

“Despite the fact that children have a lot to learn in a short time...teachers should prioritise physical activity for them. Physical activity boosts children's health and confidence.” p19.

Some Headteachers believe PE is overemphasized since students need to study math and English more to achieve high exam results, which affects the school's quality rating.

“Our teachers complain that they can't fit everything into the curriculum ... why can't PE be cancelled if kids aren't doing well in math and English?... If 25% of the kids are obese, it won't be classified as underachieving, but if 20% are underachievers, it will...p1

There was a discrepancy between participants' responses regarding the school curriculum on healthy eating. Some headteachers explained that healthy eating is integrated into the science curriculum, while others were unsure.

“Schools' priority is teaching reading and writing, then whatever follows. It's hard to tell if children are taught healthy eating... Usually, math and English are assessed, not science or nutrition.”P3

4. Role models at school

Not all schools have teachers who actively educate children about healthy diets. Some schools encourage parents to pack nutritious lunches and participate in physical activities. Teachers at some schools encourage healthy eating by commenting on children's food choices and giving incentives to those who eat well. However, headteachers are concerned that this might indirectly identify obese children, parents, and staff.

“I hold running competitions to get pupils interested... but not all schools have active teachers because they're not recruited for healthy diets... If teachers are overweight and inactive, kids won't listen to their advice…"P8

" Get teachers to eat healthy to be positive examples... praise and reward children who eat fruit instead of sweets."P15

5. Partnership with Parents

Children copy their parents' lifestyles and eat and do what they are taught by their parents, therefore, parents should be included in school interventions to prevent childhood obesity. The lack of parent awareness and incorporation makes school-based intervention difficult.

“In winter, some parents worry that children will catch a cold if they exercise outside... The policy prohibiting juice at school upsets some parents because some children don't drink enough water and worry about being dehydrated.... If parents don't understand why we do that, it makes things hard...some headteachers don't take action..”P14

In addition, schools were reluctant to involve parents in obesity prevention because school-based approaches may be perceived as criticism of parental lifestyles. Most headteachers suggested that community leaders should raise parents' awareness.

“Most parents of overweight children are busy, financially challenged, or obese... Until parents cooperate, this cycle will not change... When schools ask parents what to eat and do, they think we criticize them... peer pressure or community leaders.” P19.

Headteachers highlighted school-based activities that involve parents in promoting healthy lifestyles.

“Sometimes we invite parents to school meals with their children to introduce healthy food options...” P25.

“Every first Wednesday there was a cooking club at the community center... This project lasted four years, but stopped after COVID-19.”*P13*

Parents' limited availability has challenged schools' partnership with parents during school hours.

“Despite our efforts, parents rarely attend our activities since they are busy working, and workshops can't be held after 4:00 pm or on weekends.”P11

6. Extra-curricular activities on healthy diets and physical activities

Schools often organize before or after school activities such as sports and cooking clubs, charging between £1 and £10 per session, which typically last 40–60 min. These fees are intended to compensate teachers and encourage students' dedication. However, the cost of these clubs can put children who are not eligible for benefits at a disadvantage.

“Schools offer breakfast clubs, sometimes for free, to ensure pupils have something healthy to eat before school starts.”P6

“For after-school cooking clubs, teachers choose the recipes...kids bring ingredients or we buy them from club fees...kids cook at school and take food home...for safety reasons, we don't fry in school, so we cook healthy stuff.”P29

“It's just not possible for us to keep our clubs running without parents paying... Free classes may lead to no one showing up, especially in the winter. We've been told it's expensive, but we can't make it for free. It's not in our budget. £1.50 for 40 minutes...” P18

7. School capacity and resources

PE is not available to girls from conservative backgrounds in schools in deprived areas because of limited clothing exchange facilities. Since COVID-19, many schools have required children to attend school in PE clothes on PE days, which has led to girls' participation in PE.

“33 pupils must go to the exercise hall, so privacy is difficult to maintain... Most girls don't do exercises or wear PE clothes under their uniforms and put them on afterwards, so it's not hygienic... After COVID-19, kids started wearing PE clothes all day. But is that healthy?”P14

Almost all schools have a school nurse who checks children's weight annually and reports that to parents. Some schools, especially those in deprived areas, check children's weight less frequently since their school nurse is responsible for many other schools that are far apart.

“We count on school nurses to educate parents and children...due to fund restrictions one nurse looks after many schools, so they can't do it”P4

Several headteachers noted that schools require additional funding for obesity interventions, particularly parental involvement and extracurricular activities.

“A school's resources are limited, especially after COVID... We have had to ask for donations to upgrade our kitchen, purchase equipment, shelves, and boxes... We cannot keep asking for donations; a fund must be established...Parents living in deprived areas cannot always donate.” P1

"Qualified parents sometimes volunteer, but they also have other commitments. We need PA coaches with experience, but who will pay them? We can hire a health visitor to monitor children and educate them about healthy eating and lifestyles, but at a cost...” P16

Local councils should provide more support to headteachers in shaping school policies on healthy eating and involving parents.

“We have heard the council intends to establish restrictive zones around schools to limit access to unhealthy foods, but we haven't been formally informed. The biggest problem is that other schools and government bodies aren't collaborating."P24.

### Barriers and facilitators

The identified barriers and facilitators of implementing obesity prevention interventions in schools are summarized in Table 2.

**Table 2:** Barriers and facilitators of implementing obesity prevention interventions in West Yorkshire Primary Schools, UK

Facilitators	Barriers
Existing national school policies that promote healthy eating at school	Headteachers underestimate childhood obesity and misperceive obesity prevention
Offer healthy food at school (breakfasts, lunch, snacks)	Limited clothes exchanging space at some school
School meal portion sizes can be controlled	Time constraints and a busy school curriculum
All students in the first three grades(Key-stage-1)were offered free school meals	Overloaded school nurse
School curriculum includes mandatory physical education sessions	Only poor children are entitled to free school meals
Teachers are trained and qualified to teach physical education	Poor monitoring of meals eaten at school
Schools have space and equipment for physical activities	School meals taste and are prepared differently from what children are used to
Wearing PE clothes from home	Only parents control packed lunch contents and portion sizes
School nurses weigh children regularly and inform parents	Poor parents’ awareness about healthy diet and physical activities
After-school clubs teach children how to cook healthy recipes	Parents too busy to attend school activities
Teachers, parents, and children can serve as role models at school	Not all schools have active teachers to be role models
Recognizing and rewarding children who eat healthily at school	Limited school funds
Staff and headteachers' commitment and leadership	

## Discussion

The study aimed to explore the current role of primary schools in preventing childhood obesity, highlighting the barriers and facilitators from the perspective of head teachers in West Yorkshire, UK. We found that some school policies, activities, and curricula are already in place to prevent childhood obesity. Schools, however, implement and respond to these policies and activities differently depending on the schools' circumstances, staff, and resources, see The study findings are consistent with other studies' findings at the country level [19].

Reviews of current UK school policies on obesity prevention revealed that with the redesigned National Curriculum and revised assessment procedures schools spend more time and effort to reach the expected education standards. As a result, extracurricular activities that encourage PE and raise awareness were significantly reduced and it became difficult for the school to implement, sustain, and strengthen extracurricular activities that encourage PA and healthy eating (Pineda et al., 2019, Griffin et al., 2021).

A key finding of the current study was that headteachers acknowledged parents' dual role in childhood obesity as they contribute to the problems but are also important partners in implementing solutions. Partnership with parents increases the effectiveness of childhood obesity preventive interventions in various settings in the UK [19, 30], particularly among certain ethnicities and cultures [15, 28]and elsewhere [37].

Barriers and facilitators identified in the study (Table 2) highlight the complex relationship between obesity-related behaviors (unhealthy eating and physical inactivity) and social inequity. For example, many steps are taken to ensure healthy school meals are provided. Apart from the first 3 years, which are free for all children, free meals are provided to only eligible children. Despite being ineligible for free school meals, many families with multiple children cannot afford to pay for meals at school. Also, schools in deprived areas have limited access to donations and receive less support for extracurricular activities that are designed to encourage physical activity and healthy eating. Other studies from the UK and other countries have also highlighted health inequities associated with childhood obesity [16, 33, 34].

Staff, especially headteachers, play a crucial role in schools' engagement with obesity prevention interventions. However, a significant barrier is the shortage of school personnel, particularly school nurses, whose numbers have declined by 35% over the past five years due to poor long-term investment [18]. This has led to eliminating their role altogether in some areas; despite their crucial role in enhancing children's health and nutritional status [13, 32].

The findings also highlighted headteachers' views that restrictions in school funding hinder the expansion of curricular school activities and the implementation and sustainability of extracurricular school activities. Through an online search, the researcher identified governmental funds that schools could use to improve their physical education activities [4], but the participants in this study were unaware of these funds. Better communication and guidance are required to increase headteachers' awareness and ability to apply for and obtain this funding.

The clinical relevance of this study lies in its potential to inform the design and implementation of effective school-based obesity prevention programs, addressing the growing burden of childhood obesity—a critical public health issue linked to conditions such as type 2 diabetes, cardiovascular diseases, and psychological health concerns. By identifying practical barriers and enablers, the research provides actionable insights for policymakers, educators, and healthcare providers, guiding multidisciplinary collaborations to design interventions that are feasible, impactful, and sustainable. These findings can be translated into practical frameworks for intervention design, evaluated for effectiveness in diverse school settings, and leveraged to explore the role of policy changes and stakeholder partnerships in overcoming systemic challenges, ultimately contributing to reduced obesity rates and long-term clinical burdens.

## Strengths and limitations

The study's strength is its comprehensive analysis of head teachers' perspectives on what needs to be considered in order to enhance the role schools can play in preventing childhood obesity. However, the study has limitations, including the fact that the study sample was not randomly selected and was limited to West Yorkshire; thus, the findings cannot be generalized to all primary schools in the UK. In addition, the findings may be biased by the participants' perspectives, as children safeguarding regulations at school didn't allow the researcher to use observational methods. However, incorporating heterogeneity, such as differences in age and years of experience managing rural and urban schools, into the recruitment process of the 32 headteachers may help mitigate individual biases and improve the generalisability of the findings.

## Conclusion

We identified the current role of primary schools in preventing childhood obesity and highlighted the barriers and facilitators from the perspective of head teachers in West Yorkshire, UK. Implementing policies and curricular and extracurricular activities to prevent childhood obesity varies depending on schools’ resources, staff and headteachers' beliefs and attentiveness, and parents' awareness regarding childhood obesity. The findings emphasise the critical need for collaborative efforts among all stakeholders, including families, teachers, policymakers, and healthcare professionals. External partners and organisations, like school nurses, should help develop and deliver training and guidance for parents and staff. To better control food consumption at schools, all primary school pupils should be eligible for free school meals. Schools may receive government funding for this but should receive guidance and training on applying for and obtaining the financing.

The study offers valuable insights for policymakers, educators, and public health practitioners who aim to design and implement effective interventions in the school setting to prevent childhood obesity. It provides a foundation for improving school-based strategies that indirectly contribute to better health outcomes for children. The study highlights that effective obesity prevention requires a multi-level approach integrating school-based initiatives with family and community involvement. This indicates a need for future research to explore multi-level interventions that combine the efforts of schools, families, and communities to address barriers and enhance the sustainability and impact of these programs.

## Data Availability

No datasets were generated or analysed during the current study.
